# Cyclophilin D: An Integrator of Mitochondrial Function

**DOI:** 10.3389/fphys.2020.00595

**Published:** 2020-06-17

**Authors:** Georgios Amanakis, Elizabeth Murphy

**Affiliations:** Cardiovascular Branch, NHLBI, National Institutes of Health, Bethesda, MD, United States

**Keywords:** cyclophilin D, permeability transition pore, mitochondrial function, ATP synthase, cyclosporine A, peptidyl-prolyl cis-trans isomerase

## Abstract

Cyclophilin D (CypD) is a mitochondrial peptidyl-prolyl cis-trans isomerase, well-known for regulating the mitochondrial permeability transition pore (PTP), a nonspecific large conductance pore whose opening leads to cell death and has been implicated in ischemia/reperfusion injury in multiple organs, in neurodegenerative disorders, and in muscular dystrophies. While the main target of CypD is a matter of ongoing research, inhibiting CypD protects in models of those diseases making it an interesting therapeutic target. The present review focuses on post-translational modifications of CypD that have been identified by recent studies, which can alter the regulation of the PTP and contribute to understanding the mechanisms of action of CypD.

## Introduction

**Figure 1:**
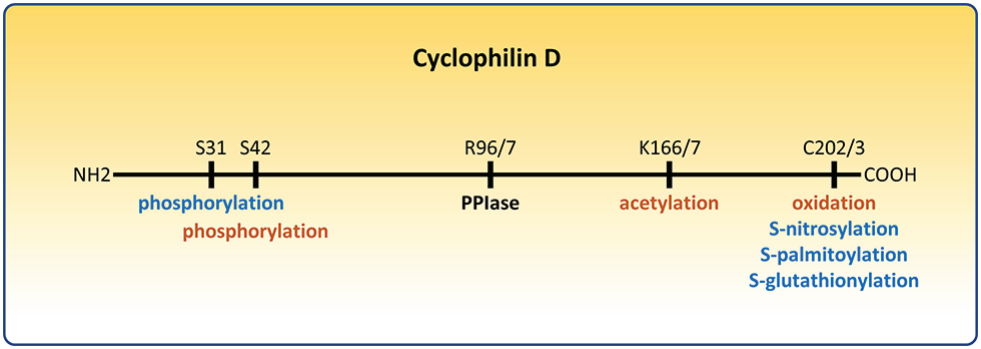
Post-translational modifications of cyclophilin D (CypD) that affect the permeability transition pore (PTP). Modifications that sensitize the PTP are depicted in orange. Modifications that desensitize the PTP are depicted in blue. R96/7, K166/7, and C202/3 represent homologous arginine, lysine, and cysteine residues in the mouse/human form of CypD, respectively.

**Figure 2:**
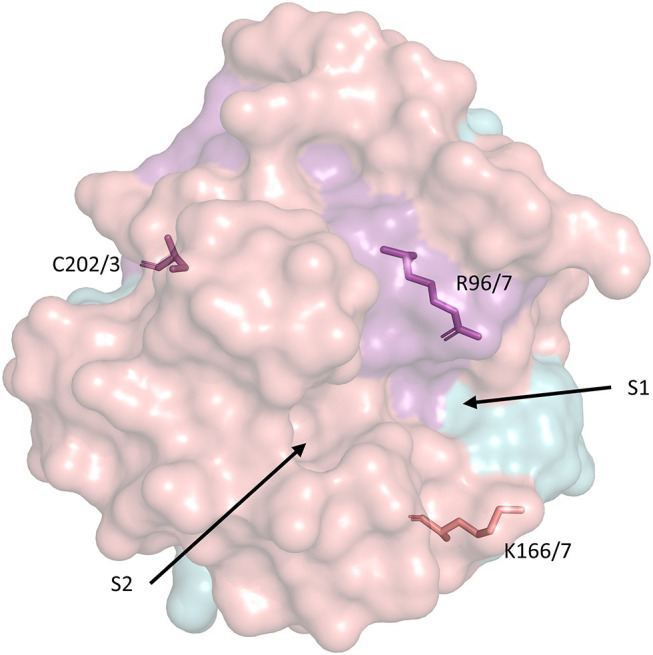
Surface representation of the secondary structure of human CypD with key residues related to PTP activation and catalytic activity. S1 and S2 represent the PPIase catalytic pockets (Davis et al., 2010). The model was retrieved from the Protein Data Bank (PDB ID: 3QYU) and rendered using PyMol (Schrodinger LLC, 2015). R96/7, K166/7, and C202/3 represent homologous arginine, lysine, and cysteine residues in the mouse/human form of CypD, respectively. Serine residues 31 and 42 (identical in mouse/human CypD) are not included in any currently available CypD model.

Cyclophilin D (CypD) is a highly conserved peptidyl-prolyl cis-trans isomerase (PPIase) that plays an important role in mitochondrial biology. It is encoded by the genomic Ppif gene and contains a mitochondrial targeting sequence which is cleaved upon entering the mitochondrial matrix, reducing its size from 22 to 19 kDa. It was named, like all cyclophilins, after its ability to bind the drug cyclosporine A (CsA) (Walsh et al., 1992). Cyclophilins, a family with more than 15 members, have been shown to act as chaperones accelerating protein folding and maturation, as well as playing a critical role in signal transduction and the immune response. Although the physiological role of CypD remains elusive, CypD has been shown to be a sensitizer of the permeability transition pore (PTP), a nonspecific large conductance pore whose opening leads to dissipation of the inner mitochondrial membrane (IMM) potential, loss of ATP production, and eventually cell death (Bauer and Murphy, 2020). The PTP has been implicated in ischemia/reperfusion (I/R) injury in the heart (Lim et al., 2011; Bibli et al., 2019), brain (Uchino et al., 2002; Schinzel et al., 2005), and kidney (Park et al., 2011; Yang et al., 2019), in neurodegenerative disorders (Warne et al., 2016), and in muscular dystrophies (Pato et al., 1983; Dubinin et al., 2020). CypD has been reported to interact with the F1F0-ATP synthase (Giorgio et al., 2009; Bonora et al., 2017), the phosphate carrier (PiC) (Leung et al., 2008), and the adenine nucleotide translocator (ANT) (Kokoszka et al., 2004; Karch et al., 2019), all of which have been proposed as potential components of the PTP. While irreversible opening of the PTP is associated with cell death, short transient openings may have a role in modulating matrix calcium (Petronilli et al., 1999; Bernardi and von Stockum, 2012; Lu et al., 2016; Agarwal et al., 2017), which in turn can regulate mitochondrial bioenergetics (Elrod et al., 2010; Glancy and Balaban, 2012; Tarasov et al., 2012). Thus, CypD as a sensitizer of the PTP may affect mitochondrial bioenergetics, and this may provide insight into its physiological function. Interestingly, the ATP synthase can oligomerize with ANT and PiC to form a synthasome to increase the efficiency of ATP production and its translocation to the cytosol, and CypD has been reported to regulate its assembly (Beutner et al., 2017). The initial studies to elucidate the role of CypD involved inhibition, deletion, and overexpression of its encoding gene (Baines et al., 2005; Nakagawa et al., 2005; Devalaraja-Narashimha et al., 2009; Elrod et al., 2010; Menazza et al., 2013). Recent work revealed CypD can undergo many different post-translational modifications (Linard et al., 2009; Hafner et al., 2010; Kohr et al., 2011b; Nguyen et al., 2011; Sanchez et al., 2011; Bochaton et al., 2015; Parks et al., 2018; Amanakis et al., 2020), including oxidation, S-nitrosylation, S-palmitoylation, S-glutathionylation, phosphorylation, and acetylation. The present review will focus on CypD as an integrator of mitochondrial physiology and pathophysiology through its post-translational modifications (Figures 1, 2).

## Cypd Modulates Mitochondrial Function

Mitochondria are the power house of the cell; they create energy in the form of ATP through oxidative phosphorylation. The electron transport chain (ETC, complexes I–IV) uses reducing equivalents from fatty acid oxidation, glycolysis, and the Krebs cycle to transfer electrons ultimately to oxygen, pump protons from the matrix to the intermembrane space, and thus generate an electrochemical gradient across the inner mitochondrial membrane. The ATP synthase exploits this gradient to create ATP from ADP and inorganic phosphate. Inorganic phosphate is imported from the cytosol by the PiC. The newly produced ATP however is localized in the matrix and needs to be transferred to the cytosol for the cell to use it. This transport is mediated by the ANT, which also imports ADP from the cytosol. To increase the efficiency of ATP production and transportation to the cytosol, the ATP synthase can oligomerize with the ANT and PiC, forming synthasomes (Beutner et al., 2017). Furthermore, the ETC can form super complexes to increase the efficiency of proton gradient generation (Acin-Perez and Enriquez, 2014; Milenkovic et al., 2017). Transport of electrons by the ETC and consumption of oxygen by cytochrome oxidase, the final electron acceptor, are coupled to ATP production by the ATP synthase. In well coupled mitochondria, electrons are not transported and oxygen is not consumed unless ADP is present. Mitochondrial coupling is greatly dependent on the permeability of the IMM. If there are leaks across the IMM (uncoupled mitochondria), the electrochemical gradient is dissipated which leads to less efficient ATP production. The PTP (Bauer and Murphy, 2020) provides a means to directly regulate the conductance of the IMM and mitochondrial coupling as its opening dissipates the proton gradient and uncouples electron transport from ATP production.

CypD is a regulator of the PTP, and thus of mitochondrial coupling. It sensitizes PTP to calcium and oxidative stress. It can also directly bind to the ATP synthase and reduce ATP production while its displacement from the inner mitochondrial membrane by CsA increases ATP synthesis (Giorgio et al., 2009). In heart, brain, and liver mitochondria, CypD also regulates the assembly of synthasomes (Beutner et al., 2017). Their formation is stimulated by respiration while their disassembly favors PTP opening. Interestingly inhibiting or deleting CypD increases the stability of the synthasomes, suggesting a model in which CypD binds to and limits their assembly (Beutner et al., 2017). However, from the data provided it is unclear whether this is a direct effect of CypD or if matrix calcium levels, redox state, or inner membrane potential are also involved.

The PPIase activity of CypD may also have a regulatory role on the PTP. In a study employing CsA analogues, inhibition of PTP correlated well with inhibition of PPIase activity of CypD (Nicolli et al., 1996). The arginine at residue 96 (R96, homologous to R97 in human CypD) is important for PPIase activity and mutating it to a glycine abolishes the PPIase activity of CypD. CypD^−/−^ fibroblasts are protected against oxidative stress; however, consistent with the previous study, the protection is lost in cells expressing a wild type (WT) CypD but not in cells expressing the R96G CypD mutant (Baines et al., 2005). These findings suggest that the PPIase activity of CypD facilitates PTP activation. However, the exact mechanism is unknown. Cis-trans prolyl isomerization of PTP components may expose binding sites to calcium which would affect the sensitivity of PTP to it. It has also been suggested that CypD activates the PTP independent of its PPIase activity (Scorrano et al., 1997). Future studies will be needed to address the role of the PPIase activity in regulating the PTP. Interestingly, the PPIase activity normalized to the expression level of the ATP synthase was associated with changes in synthasome assembly, leading to the conclusion that increased synthasome assembly with the ATP synthase in high order oligomers decreases the probability of PTP formation from ATP synthase monomers or dimers (Beutner et al., 2017). However, differentiating between independent effects of CypD and those resulting from uncoupling due to CypD-mediated sensitization of the PTP is challenging. Furthermore, it is unclear how post-translational modifications of CypD affect those functions.

CypD can regulate the expression of mitochondrial genes, thus affecting cell proliferation and differentiation (Radhakrishnan et al., 2015). It interacts with mitochondrial transcription factor and regulates mitochondrial RNA synthesis of subunits of the NADH dehydrogenase (ND1), cytochrome c oxidase (COX1), and ATP synthase (ATP6). Furthermore, deletion of CypD has been shown to activate mitochondrial retrograde signaling leading to transcriptional changes in gene expression, modulation of a chemokine/chemokine receptor signature, and activation of the inflammatory mediator STAT3 that affects cell motility and proliferation (Tavecchio et al., 2013).

Loss or inhibition of CypD has been shown to increase mitochondrial calcium which activates the mitochondrial dehydrogenases and shifts the metabolism from fatty acids to glucose (Elrod et al., 2010). Additionally, the increase in NADH/NAD^+^ ratio results in inhibition of deacetylases and increases acetylation of proteins (Nguyen et al., 2013) comprising pathways involved in branched chain amino acid metabolism, pyruvate metabolism, and the Krebs cycle (Menazza et al., 2013). Interestingly, CypD has been reported to be essential for the activity of the trifunctional protein, a central enzyme in β-oxidation of fatty acids (Tavecchio et al., 2015). Consistent with the metabolic shift to glucose, CypD^−/−^ mice were more susceptible to heart failure compared to WT. These results indicate that CypD has a central role in controlling mitochondrial bioenergetics by regulating matrix calcium. It is widely known that failing hearts exhibit a metabolic shift from fatty-acid to glucose oxidation (Karwi et al., 2018). However, the role of CypD in heart failure is unclear and future research should focus on how to exploit it as a therapeutic target in this disease.

## Oxidation/S-Nitrosylation/S-Glutathionylation/S-Palmitoylation

Oxidative stress can modify structural and functional properties of proteins, and cysteine residues are of particular interest due to their reactive thiol group. Oxidation of cysteines of CypD influences the conformation of the enzyme and its activity (Linard et al., 2009). Site-directed mutagenesis allowed the identification of cysteine 203 of human CypD (homologous to cysteine 202 in mice, abbreviated as C202/3) as an important redox-sensitive residue. When C202/3 is oxidized it can form a disulfide bond with C157 which decreases its PPIase activity by 20% (Linard et al., 2009). As discussed, it is unclear whether PPIase activity is essential for PTP activation. Additional experiments are needed to address this issue. Previous studies have also shown that C202/3 undergoes S-nitrosylation in ischemic preconditioning, a cardioprotective strategy consisting of repetitive non-lethal I/R cycles (Kohr et al., 2011a,b). The mutation of C202/3 to a serine or treatment with a nitric oxide donor in mouse embryonic fibroblasts desensitized the PTP in oxidative cell death (Nguyen et al., 2011). In addition, tachycardic preconditioning desensitizes the PTP and increases S-glutathionylation of CypD (Sanchez et al., 2011). These findings suggest that S-nitrosylation and S-glutathionylation shield C202/3 from further oxidative damage and are thus cardio-protective. C202/3 also matches an S-palmitoylation motif common in soluble proteins (Collins et al., 2017). Recent data from our lab show that CypD is abundantly S-palmitoylated at C202/3, and that increased matrix calcium during ischemia leads to de-palmitoylation of C202/3, allowing it to undergo oxidation and to interact with the PTP (Amanakis et al., 2020). Of note, S-palmitoylation, S-nitrosylation, and the mutation of C202/3 to a serine do not affect the PPIase activity of CypD (Nguyen et al., 2011; Amanakis et al., 2020). In summary, oxidation on C202/3 sensitizes the PTP while S-nitrosylation, S-glutathionylation, and S-palmitoylation confer protection.

## Phosphorylation

The effects of phosphorylation of CypD differ according to the phosphorylated residue. Glycogen synthase kinase-3 (GSK-3) has been reported to phosphorylate CypD and to enhance PTP opening and, consistent with this concept, GSK-3 inhibition (which would decrease CypD phosphorylation) protects from PTP opening in human cancer cell models (Rasola et al., 2010). Similarly in a rat model, treatment with an inhibitor of GSK-3 decreased CypD phosphorylation and its association with ANT and prevented PTP opening which decreased I/R injury (Teodoro et al., 2018). The site of phosphorylation by GSK-3 was not identified in these studies, and it is unclear how GSK-3 localizes to the mitochondrial matrix since it lacks a mitochondrial targeting sequence (Rasola et al., 2010). In contrast to these studies, activation of the PI3K pathway resulted in Akt2 mediated phosphorylation of serine 31 (S31) of CypD in tumor cells. In this study the mutant S31A CypD, which cannot be phosphorylated, when expressed in CypD^−/−^ cells lacked PPIase activity, exhibited defective mitochondrial bioenergetics with reduced glucose utilization, impaired oxygen consumption, and decreased ATP production and showed findings in accordance with sensitization of the PTP such as loss of membrane potential, discharge of cytochrome c, and reduced cell viability (Ghosh et al., 2015). Finally, it was reported recently that the germline deletion of the mitochondrial calcium uniporter results in increased CypD phosphorylation at serine 42 (S42), increased association of CypD with the ATP synthase, a proposed component of the PTP, and PTP sensitization to calcium (Parks et al., 2018). The kinase responsible for phosphorylation of S42 was not identified. Summarizing phosphorylation on S31 has been shown to be required for normal mitochondrial bioenergetics and cell viability while phosphorylation on S42 has been associated with PTP sensitization. Notably, since the site of phosphorylation was not identified in studies involving GSK-3 mediated phosphorylation (Rasola et al., 2010; Teodoro et al., 2018), there could be additional serine residues whose phosphorylation regulates the PTP. Thus, depending upon the site of phosphorylation, it appears to have different effects on CypD stimulation of PTP. Interestingly, PI3K increases phosphorylation of AKT, leading to phosphorylation and inhibition of GSK-3, which both lead to inhibition of the PTP. These findings suggest that AKT and GSK-3 phosphorylate different residues on CypD and are consistent with cardioprotection conferred by activation of the PI3K pathway (Tong et al., 2000, 2002).

## Acetylation

CypD alters the cardiac mitochondrial acetylome and has been shown to be a substrate of the mitochondrial deacetylase sirtuin 3 (SIRT3) (Nguyen et al., 2013). Lysine 166 (K166, homologous to K167 in human CypD) has been reported to be deacetylated by SIRT3 while in SIRT3^−/−^ mice CypD is hyperacetylated at this residue, leading to sensitization of the PTP and increased cell death in a model of transaortic constriction (Hafner et al., 2010) In addition, heart failure induced by coronary artery ligation in a rat model decreased the expression of SIRT3 and increased CypD acetylation. In line with these findings, another report showed that hypoxia in cardiac myoblasts also increased CypD acetylation while overexpression of SIRT3 attenuated this effect and was protective against cell death (Bochaton et al., 2015). Of interest, expressing the CypD acetylation mimic K166Q decreased calcium retention capacity and increased cell death in mouse embryonic fibroblasts, while the non-acetylated CypD mimic K166R had the opposite effects (Bochaton et al., 2015). In the same study, *in vivo* I/R injury increased CypD acetylation while ischemic postconditioning decreased infarct size and cardiac CypD acetylation but failed to protect in SIRT3^−/−^ hearts. Similar effects of I/R injury in the heart and brain were attenuated by hypothermia and CypD inhibition in a rabbit model (Jahandiez et al., 2017). These data suggest that acetylation of CypD on K166 promotes cell death by sensitizing the PTP and that the protection with ischemic postconditioning is dependent on SIRT3 deacetylation of this residue. Notably, the acetylation of mitochondrial proteins is dependent on acetyl CoA abundance (Baeza et al., 2015; Weinert et al., 2015) and this may alter CypD acetylation, thereby altering PTP and perhaps regulating the synthasome assembly.

## Inhibition of Cypd Dependent Ptp Opening with Cyclosporine a and Translation To the Clinic

There are a large number of studies showing that CypD is a regulator of the PTP, an activator of cell death in I/R injury and that cyclosporine A desensitizes PTP and confers cardioprotection (Weinbrenner et al., 1998; Hausenloy et al., 2002; Argaud et al., 2005; Lim et al., 2007). However, the translation of these findings to the clinic has failed (Cung et al., 2015; Ottani et al., 2016). The reasons proposed for the lack of translation include the incomplete understanding of the cell-death pathway involving the PTP, the possibility that cyclosporine A did not reach the mitochondria in the first minutes of reperfusion, and the differences in comorbidities and co-medications between our patients and the experimental models (Heusch, 2017; Botker et al., 2020). Another possibility is that post-translational modifications of CypD might occur with comorbidities, co-medications, or aging, thus altering the regulation of PTP. Interestingly, there is evidence of increased CypD binding to the ATP synthase and decreased ATP synthase activity in the brain of aging mice (Gauba et al., 2017). The cause for the increased interaction might be a post-translational modification on CypD. In this case, it is unclear whether CsA would interact with CypD already bound to the ATP synthase and, thus, if it would protect in these mitochondria. In addition, the failure to translate may be due to the inhibition of a physiological function of CypD. As discussed, chronic genetic loss of CypD results in cardiac failure in response to both physiological and pathological stimuli, possibly suggesting a necessary function beyond cell death regulation (Elrod et al., 2010). A more complete understanding of the physiologic function of CypD and PTP mediated cell death is mandatory before pursuing CypD dependent PTP’s as a therapeutic target.

## Conclusion

CypD can undergo multiple post-translational modifications and regulate mitochondrial bioenergetics and the PTP. However, most post-translational modifications of CypD have been studied in relation to the PTP. It is unclear how the majority of those post-translational modifications affect mitochondrial bioenergetics. In addition, although CypD sensitizes the opening of the PTP to stressful stimuli and possibly acts as an uncoupler by interacting directly with the ETC and the ATP synthase, its physiological role remains elusive.

## Author Contributions

GA wrote initial draft of the manuscript. EM edited the manuscript.

## Conflict of Interest

The authors declare that the research was conducted in the absence of any commercial or financial relationships that could be construed as a potential conflict of interest.
